# On the Employment of Ether in Surgical Operations

**Published:** 1847-05

**Authors:** 


*On the employment of Ether in Surgical Operations.* By the Editor of the London Medical Gazette.

The burst of enthusiasm which has ushered in the use of ether as a means of rendering persons unconscious of pain, while under surgical operations, having had time to extend itself through the length and breadth of our land, it becomes us, as journalists, to consider calmly whether our present experience of the influence of this new agent is really confirmatory of, or in opposition to, the glowing paragraphs almost daily found in our newspapers :—whether, in fact, the introduction of this medicine, for the purpose alluded to, has been productive of the unmixed good that the public have been led to believe,—and whether, in estimating its value, medical men have followed that discreet, philosophical, and respectable course which might have been expected from the members of a liberal profession.

It is a well known fact, that intoxication, whether produced by ordinary potations, by opium, by Indian hemp, or indeed by many other substances, does, when it is carried far enough, produce in the system a state marked by a more or less complete unconsciousness of pain ; and this, with respect to opium and hemp, whether taken into the stomach, or when introduced into the lungs by smoking. The same, or a similar condition, is induced by the use of ether. It is true we have not been accustomed to introduce ether into the stomach for the purpose of inducing intoxication, previous to the performance of surgical operations ; but we are by no means convinced that its effects would not be equally remarkable were it so applied, or that the plan might not be accompanied with less risk, than when it is introduced into the system through the agency of the lungs. While the stomach is modified for the reception of various substances, the lungs are organised for the introduction of atmospheric air, and it is constantly observed that the air-passages become impatient under the presence of the ether vapour, as well as under many other gaseous fluids.

Under ether intoxication the most severe surgical operations may often be performed and the patient, when the influence is dissipated, will assure those around him that he has experienced no pain, and that he has been probably under the influence of a pleasant dream. It is, however, certain that under the knife, patients thus intoxicated will struggle, or even scream, violently ; and yet, when all is passed, they will tell you that they have been totally unconscious of the proceeding which has given rise to it. It is also the fact, that many signs of exhaustion may be apparent afterwards, and they may be quite as decided as those which are observed after ordinary operations, and indeed much more than might be expected to occur after unimportant operations. How these circumstances are to be explained it is not easy to determine ; some persons suggest that it is owing to a strong preoccupation induced in the brain by the intoxication,—others maintain that the power of perception is for the time extinguished, as you stupify the brain and make it insensible to pain by an overwhelming dose of opium ; and by them the convulsive movements are referred to a reflex action. Whether any of these explanations be correct is doubtful.

The fact, however, still remains—certain indications of pain are apparent, although the patient, when the influence has ceased, is not aware of anything which could have given rise to them ; and certainly there is often as much appearance of exhaustion as might be expected from the apparent amount of suffering.

These facts being admitted, it may next be asked—Can we determine in advance what dose of the vapour the patient may require to produce the wished-for effect, as we can determine what dose may be required by certain remedies to produce a given effect ?—(as we know, in fact, that in nineteen cases out of twenty a scruple of jalap will purge—a scruple of ipecacuanha will vomit.) Certainly not ; for if we take two similar kinds of apparatus, with a like quantity of similar ether in each, of the same temperature, and let it be inhaled by two patients with the same rapidity,—in one the desired effects may be obtained within five minutes ; in the other it may be not for a quarter of an hour ; and there is no certain sign by which we can be assured that the patient has had enough. The state of the pupil and the state of the pulse are extremely variable, and therefore not to be relied on ; probably the best test is the change in the breathing. Then it also happens with ether, as with opium, or, indeed, any species of intoxicating substance, that the form which the intoxication may assume cannot with any certainty be predicated. One man, under opium smoking, will become stupidly insensible—almost comatose ; another will become furious and "run a muck," as is often observed in the East. Under ether, probably one case in twenty will be accompanied by this sort of excitement.

Then, as to the operation itself, it is not always desirable that the patient should be unconscious : it is sometimes well to know how much suffering is experienced—whether nervous cords are unnecessarily interfered with, and so on. No surgeon, while opening a hernial sac, or making a section of the prostate, could regard without apprehension the chance of some convulsive movement. When a patient's senses are entire, these things do not often happen ; but during intoxication he may bring his jaws together while a cutting instrument is in his mouth.

Supposing it to be admitted, however, that the administration of ether under ordinary circumstances is a certain means of rendering a patient insensible during operation (which is by no means the case,) and supposing the dose could be exactly adapted to every case, and supposing the effects to be uniform, do we know of any evil consequences which have up to this time resulted from its use?—and are they of so serious a character as to make a prudent man hesitate in recommending his patient to become subject to its influence?

Great excitement of the nervous system, sometimes approaching to apoplexy,—an asthmatic condition of the respiratory organs,—spitting of blood,—syncope,—are among the results which have been observed; but these are by no means the worst. In many instances —already we are aware of six or seven—death has followed quickly upon operations so performed. It may be that in some of these cases death would have resulted even had the ether not been inhaled; but, as far as we have been informed, several of the deaths have occurred under circumstances which are not observed under ordinary operations. It is to be regretted that the same alacrity is not shewn in furnishing journals or newspapers with these fatal cases as with the "dexterous operations." We are sorry to say it, but we believe it to be the fact, that none of these cases, with the exception of that reported by Mr. Nunn, have been recorded in this country.

How different is the conduct of M. Jobert, the distinguished surgeon of St. Louis, at Paris. At the sitting of the Academy of Medicine, held February 16th, he stated that in two cases death after operation had occurred in his wards, and that the inhalation practised in both instances did not appear to him to have been altogether unconnected with the fatal issue.

Why there should be this want of common honesty we cannot conceive. There are few persons who do not concede the necessity of fairly trying the agent; and, if that be admitted, its failure cannot attach any reproach to the operator unless there have been a want of proper caution in the administration. So far as the effects have been at present observed, they do not justify us in condemning the agent, but they show us the necessity of grave circumspection in its employment.

There is another matter connected with this subject which presses more heavily upon us: and it is under a strong sense of duty that we raise our voice against it. We allude to the system by which this discovery has been introduced to the public. It is so inconsistent with that relf-respect which should actuate the conduct of every member of a liberal profession, that we could not be silent without a failure of the duty which we owe to those who pursue the higher path. To extend invitations to be present at operations to every layman of his acquaintance is surely not the way in which an enlightened surgeon would seek to advance the cause of science. Under this new agent, the phenomena, where females are concerned, are often so peculiar, that the opportunities of being present are sought for by some persons as means of gratifying a prurient curiosity: and what is not less to be deplored, is the fact that what is observed has the effect of influencing incorrectly the judgment of people whose infirmities may render them the subjects of operation, but cannot tend to the advancement of science.

The experiments have been in many instances so made, that the public are no longer spectators only, but judges of the propriety of the administration of the remedy;—and patients now direct that ether shall or shall not be employed, instead of allowing the practitioner freedom of action.

It is with much pain that we have observed the headlong pursuit of any opportunity for performing surgical operations,—sometimes even without urgent necessity,—because they are likely to come before the public. One day it is the section of tendons, another the administration of ether:—and we are confident that nothing tends more to shake public confidence in professional men, than the restlessness with which each new phantom is followed and abandoned.

But what is still less worthy is the system of puffing in the public papers to which the inhalation of ether has given rise. The presence of a newspaper reporter, however able in his own field, is not necessary in the operating theatre of an hospital, and he can scarcely be a competent judge of the merits of a surgical operation. A surgeon would scarcely say that an amputation was performed in a masterly manner, when the soft parts, left to cover the bone, were insufficient for the purpose, by an inch and a half, the bone being left protruding, and yet such an operation has so fascinated a reporter that he could scarcely set any bounds to his glowing description of its excellence. Is it ignorance, or something corrupt, which affords the readiest explanation of these things?

It will not do for us to wrap ourselves up in our mantles, and to inveigh against Holloway and others, if quasi respectable members of our own profession, and even Hospital Surgeons, await only a convenient opportunity to advertise themselves in terms as gross and as objectionable as those of Culverwell, Goss, and others.—*Lond. Med. Gaz.*

